# Personal correlates of creative performance: A systematic review

**DOI:** 10.3389/fpsyg.2025.1727094

**Published:** 2026-01-30

**Authors:** Jorge Cebrián, Pedro J. Ramos-Villagrasa

**Affiliations:** 1 Department of Psychology and Sociology, Faculty of Labour and Social Sciences, University of Zaragoza, Zaragoza, Spain; 2 Department of History and Social Sciences Applied to Design, Aragón School of Design, Zaragoza, Spain

**Keywords:** creative performance, creative behavior, job performance, personality, motivation, engagement, positive affect, self-efficacy

## Abstract

Creative performance drives innovation, problem-solving, productivity, and competitiveness of organizations. This research comprehensively reviews existing studies on employees’ creative job performance and integrates findings on individual factors that are associated with creative behavior. Examining 82 empirical studies of creative performance, 79 personal factors grouped into eight categories has been identified: demographic factors, personality, cognitive ability, motivation, emotions, self-efficacy, other performance dimensions, and a miscellaneous category. The most relevant personal factors associated with creative performance include educational level (*r_w_* = 0.19, 95% *CI* [.12, .27], *p* < 0.001; *I*² = 55.66%), and moderate to strong positive associations for openness (*r_w_* = 0.46, 95% *CI* [0.33,0.59], *p* < 0.001; *I*² = 93.76%), intrinsic motivation (*r_w_* = 0.39, 95% *CI* [0.28, 0.50], *p* < 0.001; *I*² = 93.37%), creative process engagement (*r_w_* = 0.52, 95% *CI* [0.23, 0.80], *p* < 0.001; *I*² = 97.39%), positive affect (*r_w_* = 0.31, 95% *CI* [0.18, 0.43], *p* < 0.001; *I*² = 81.53%), and creative self-efficacy (*r_w_* = 0.45, 95% *CI* [0.34, 0.56], *p* < 0.001; *I*² = 95.30%). The findings enhance the nomological network of creative performance, offer directions for future research, and suggest improvements for human resource processes, including employee selection, training, and performance assessment.

## 1 Introduction

Driving innovation is critical to the long-term survival of today’s organizations (Akbari et al., 2021). Creativity is the driving force of innovation and is an essential quality for a company’s long-term success (Peng and Chen, 2023). As the business environment’s competitiveness increases, professional, managerial, and administrative tasks become more complex and knowledge-intensive, and employers expect employees to become more creative (Aleksic et al., 2016). Therefore, creativity is a valuable outcome that should be cultivated and encouraged within organizations through effective human resource management practices (Villajos et al., 2019).

Creativity is a term that has avoided precise definitions owing to its inherently elusive nature, which encompasses the ability to generate novel and valuable ideas, solutions, or expressions that extend beyond conventional boundaries (Zhou and Hoever, 2023). In the work setting, being creative can be considered part of the job performance dimensions (Harari et al., 2016). When employees exhibit *creative performance*, they produce novel and potentially useful ideas about the organization’s products, practices, services, and procedures (Shalley et al., 2004).

Although prior research has analyzed how creative performance relates to various organizational outcomes, the personal factors associated with it remain insufficiently understood (Zhang et al., 2021). The assessment and analysis of creative performance as a component of job performance is essential for organizational processes, including personnel selection, compensation, and professional development (DeNisi and Murphy, 2017). This study reviews the existing literature on individual-level factors associated with creative performance, synthesizing current findings to clarify how this outcome is characterized within the field of Human Resource Management (HRM). To generate insights that inform both researchers and practitioners, the present work conducts a systematic review aimed at identifying the personal correlates of creative performance.

## 2 Job performance and creative performance

Job performance is a multidimensional construct that encompasses behaviors under workers’ control that contribute to organizational goals (Campbell and Wiernik, 2015). It is a product of individual factors, work environment, and their interplay (Yang et al., 2022). Research has consistently highlighted the multifaceted nature of performance (Lievens et al., 2021), comprising three key aspects: *task performance*, which pertains to in-role behaviors; *contextual performance* (also called Organizational Citizenship Behavior or OCB), which refers to behaviors that foster a positive organizational environment; and *counterproductive work performance*, which encompasses any negative behavior directed towards individuals or the organization. However, other domains such as *adaptive performance* (Park and Park, 2019) and *creative performance* (Zhou and Hoever, 2014) have been proposed in recent years. Organizations often underestimate the importance of creative performance for its subjective value despite its prominent role in achieving organizational success (Al-Madadha et al., 2023). This paper focuses on creative performance to emphasize the value of displaying creative-related behaviors at work.

Creative performance refers to the observable outcomes of employees’ creative behaviors, specifically the extent to which the generate ideas, products, services, or processes that meet two criteria: (1) they are original or novel, and (2) they hold potential value for or applicability within an organization (Oldham and Cummings, 1996; Zhou and Hoever, 2014). Creative performance is related to other constructs, such as creativity and innovation.

Amabile (1996) considered creativity a complex and diffuse construct arising from a combination of individual and environmental factors, resulting in the generation, development, and expression of innovative and original ideas, solutions, or artistic creations. Creativity involves thinking unconventionally and establishing connections between diverse concepts to produce something novel and beneficial (Shalley et al., 2004). On the other hand, creative performance refers to behaviors aimed at generating original ideas and innovative solutions that manifest creativity in action as the tangible result of creative thinking and problem-solving applied to organizational challenges and opportunities (Zhou and Hoever, 2023). The distinction between creative job performance and creativity is also referred to as creative performance behaviors *vs.* creative outcome effectiveness (Montag et al., 2012). In this framework, creative performance refers to the behavioral antecedent of creativity, encompassing the observable and cognitive actions individuals enact when approaching nonalgorithmic tasks within the creative process (Lubart, 2001). In contrast, creativity denotes the outcomes produced, which emerge from the interaction of individual capacities and contextual factors that support the generation of original and useful ideas (Hennessey, 2017). Regardless of the term used, it mimics the distinction between behavior and outcomes that appear in the job performance literature (Marques-Quinteiro et al., 2015). Thus, creative performance captures the behavioral expression of the creative process, whereas creativity reflects the quality of the resulting ideas (Zhou and Hoever, 2023).

Innovation, another construct related with creativity, refers to the successful implementation of new or improved ideas, products, and services or processes that transform practices, whereas creative performance focuses on the behaviors that generate these novel ideas, often seen as the initial stage of innovation (Anderson et al., 2014). Hughes et al. (2018) state that not all creative performance leads to innovation, and not all innovative behavior requires employees to be creative before they can be innovative. Therefore, the two concepts are related but not as deeply intertwined as traditionally thought.

Creative performance is key for organizations because it helps solve problems and fosters innovation, productivity, and competitiveness. Hence, more effort is needed to predict the creative performance of employees in organizations (Zhou and Hoever, 2023). By viewing creative performance as a behavioral phenomenon, we can better understand the fundamental processes and mechanisms by which employees operate. The analysis and prediction of job performance dimensions considerably impact organizational processes (Dalal et al., 2012). Following Ployhart (2021), we can outline at least three aspects: (1) it allows us to gain insights into the factors that influence productivity, efficiency, and effectiveness within a work environment; (2) research on organizational performance helps identify areas of improvement, and this knowledge can be instrumental in developing strategies to enhance employee satisfaction, reduce turnover, and boost morale; and (3) performance research is valuable for promoting employee development and well-being, as organizations can identify training and development needs, provide targeted support to employees, and foster a culture of continuous improvement.

Given the relevance of predicting creative performance, we want to explore the personal correlates of creative performance. It is necessary to understand which factors are the most relevant and how they can be categorized (Xu et al., 2018; Zhou and Shalley, 2003), because doing so may allows researchers to move beyond isolated findings and develop more comprehensive frameworks that extend the literature on the personal factors associated with creative performance.

## 3 The present study

Understanding the personal correlates of creative performance provides insights into how individual characteristics relate to innovation within organizations, as well as employee productivity and effectiveness (Anderson et al., 2014). Despite the topic’s relevance, there is no clear delineation of the personal factors related to creative performance.

To our knowledge, the main effort to combine the cumulative research on the topic is the meta-analysis conducted by Xu et al. (2018). They analyzed 22 studies and found 25 determinants of creative performance between personal factors (e.g., positive and negative affect, motivation) and contextual factors (e.g., time pressure, supervisor support). The personal factors they examined included constructs related to emotions (e.g., PANAs), motivation (e.g., intrinsic motivation), and personality (e.g., need for autonomy). They reported an overall association of 0.551 between these 25 factors and creative performance. However, they also recognized that further research is needed, particularly to examine the specific contribution of each factor.

Despite the relevance of the findings of Xu et al. (2018), we found some limitations in their study that led us to perform our own study. First of all, they reported data from workers and students, which can bias the results. Second, Xu et al. (2018) based their studies on relatively restrictive databases (i.e., the Chinese databases CNKI, Wan, and ScienceDirect and SpringerLink), which probably do not account for all creative performance research. Broader databases, like Web of Science (Wos) or Scopus probably achieve more results. Third, they only considered direct effects, leaving a gap in understanding how mediating or moderating factors may influence the overall dynamics.

Taking into account the aforementioned limitations, we propose to conduct a systematic review which: (1) analyzes the studies that report workers’ data; (2) focuses on the main current databases (WoS and Scopus); and (3) considers direct and indirect effects on creative performance.

## 4 Materials and methods

### 4.1 Inclusion criteria

Only articles published before January 2024 in peer-reviewed journals were included. There were no restrictions on participant population or geographic or cultural origin. Five criteria were established for inclusion in the review: (1) studies had to be written either in English or Spanish; (2) participants had to be workers; (3) the study design had to be quantitatively oriented; (4) the study must include a measure of creative performance; and (5) the studies had to establish a relationship between some personal construct and creative performance, where the personal construct is proposed as an antecedent of creative performance.

### 4.2 Literature search

We followed the PRISMA statement for this review (Page et al., 2021) using the Web of Science (WoS) and Scopus databases for literature research. The specific syntax used for the WoS was [“creative performance”] in the “title” field and [“work” or “job” or “organization” or “employee”] in the “topic” field. The specific syntax used for Scopus was [“creative performance”] in the “article title” field and [“work” or “job” or “organization” or “employee”] in the “abstrac” field. The search was performed in January 2024. A total of 386 articles were retrieved, of which 145 (37.6%) were duplicates.

**Figure 1:**
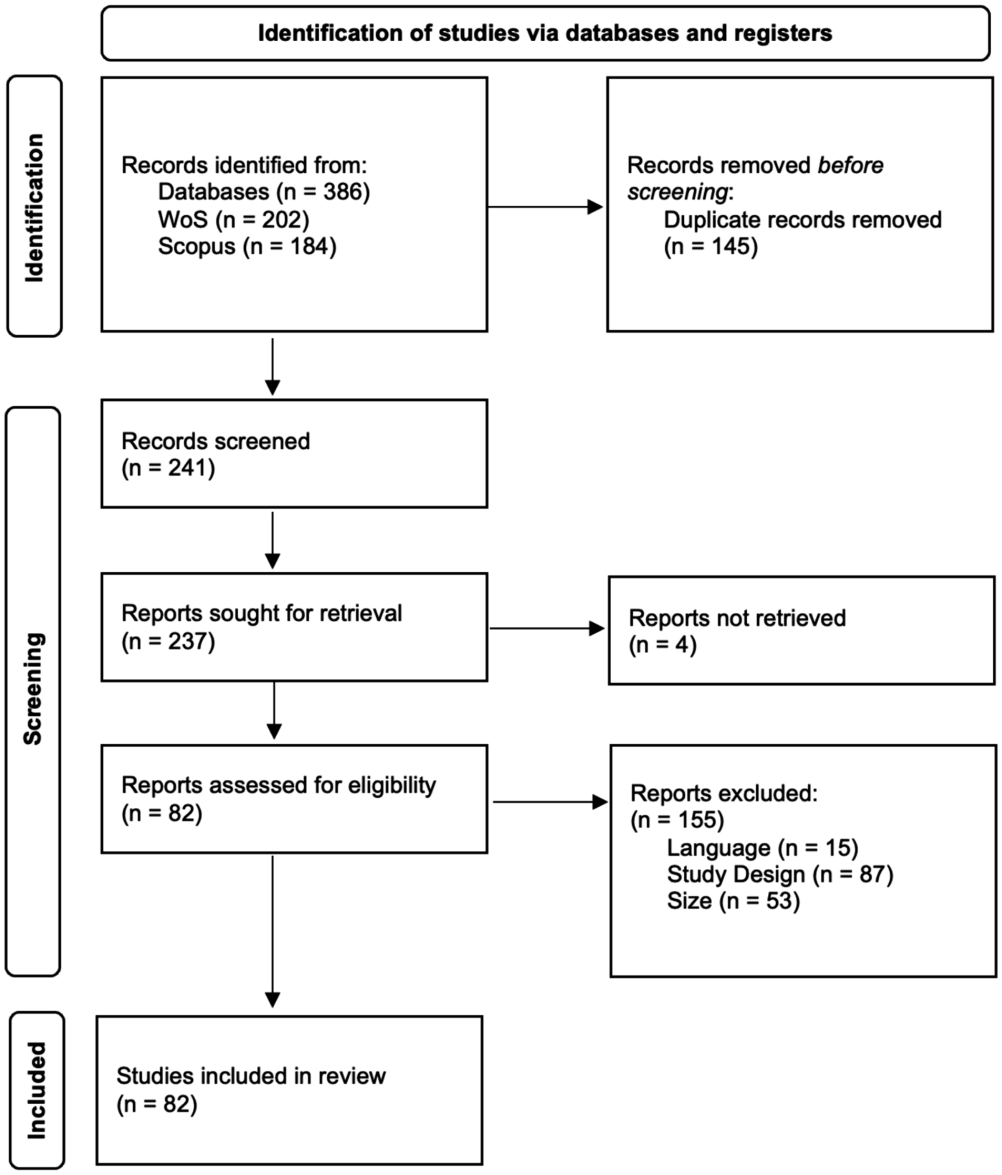
PRISMA flow diagram.

A total of 241 full-text articles were reviewed, of which 82 (34%) were included in the final study. Fifteen articles (6.2%) were excluded due to the publication language. Eighty-seven articles (36.1%) did not meet the research objectives analyzed in this systematic review (i.e., to establish relationships between personal factors and determine whether they are associated with creative performance in organizations; some articles did not measure participants’ creative performance). Fifty-three articles (22%) were excluded because the sample did not comprise employees performing a job in an organization. Four articles (1.7%) were not accessible to the research team. The PRISMA flow diagram can be seen in Figure 1.

## 5 Results

**Figure 2:**
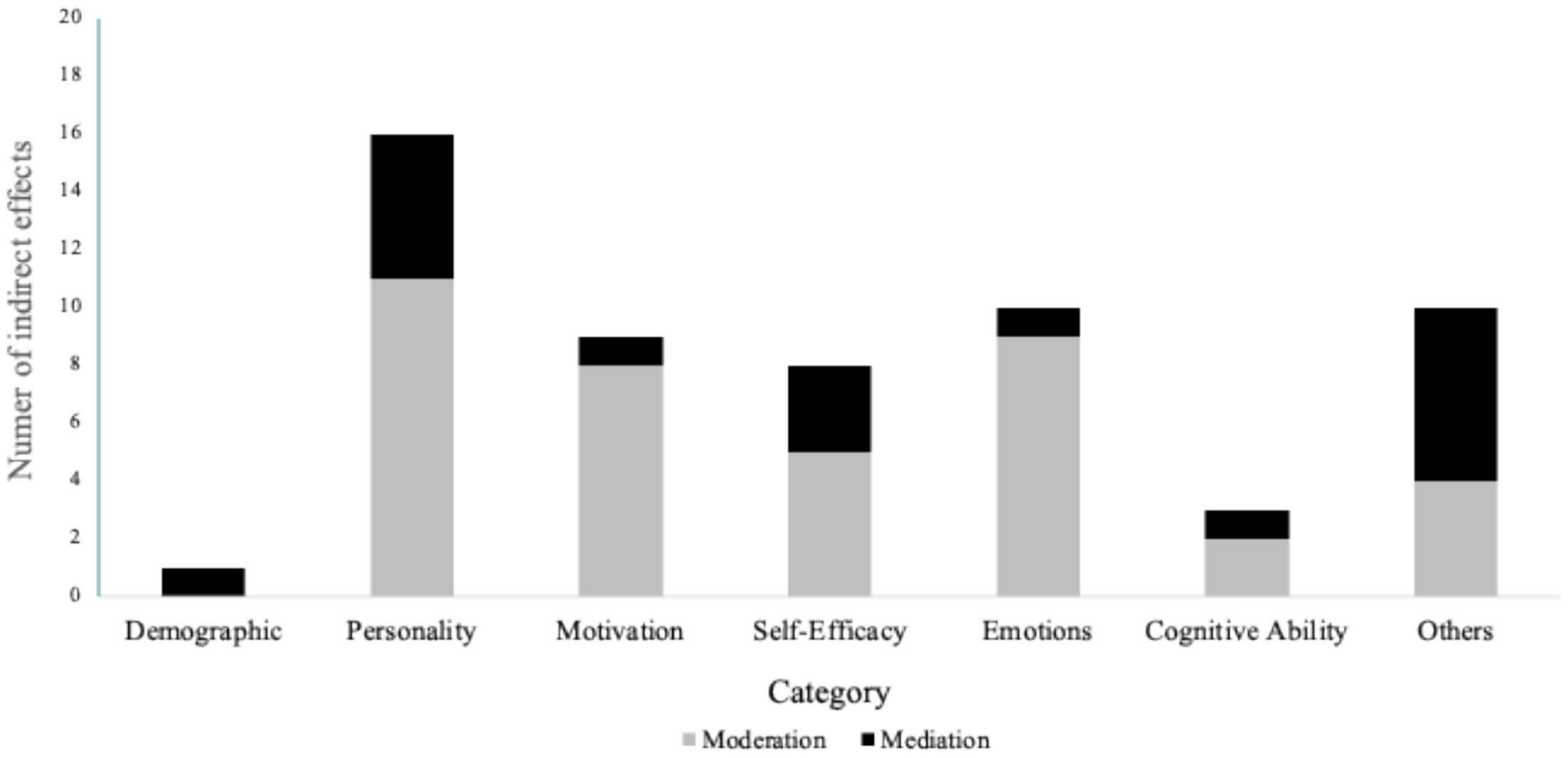
Moderated and mediated effects of personal correlates on creative performance.

Our review comprises 82 studies. Of them, the analysis revealed 79 direct effects and 57 indirect effects related to personal characteristics influencing creative performance. Of the indirect effects, most were moderations (*N* = 39; 68.4%), and the remaining were mediations (*N* = 18; 31.6%), as illustrated in Figure 2. Regarding study design, most followed a cross-sectional design (*N* = 60; 73.2%). There was consistency in the measurement of employee creative performance. Supervisor ratings were used in 44 studies (53.7%), self-reports in 35 studies (42.7%), coworker ratings in 2 studies (2.4%), and a combination of self-reports and supervisor ratings in only 1 study (1.2%). Given space limitations, indirect effects are also detailed in Supplementary material. Thus, the following sections focus on the main effects.

The personal correlates of creative performance identified in the systematic review were categorized into eight different categories: (1) demographic factors, (2) personality, (3) cognitive ability, (4) motivation, (5) emotions, (6) self-efficacy, (7) other performance dimensions, and (8) others. These categories were developed inductively after identifying the full set of personal factors across the reviewed studies, allowing conceptually similar variables to be grouped together. The amount of papers detailing each personal characteristic and the papers that support them are shown in Table 1.

**Table 1:** Personal correlates directly associated with creative performance.

Category	Construct	Reference	Country	Measurement	Industry	*N* (%F)	*r*	*p*	SD
Demographic	Gender	Aleksic et al. (2016)	Slovenia	Self-report and supervisor-report	(1) Casual sector (2) Manufacturing	129 (59%) 165 (34%)	— −0.10	— <0.05	CS
		Cheung and Zhang (2021)	China	Supervisor-report	(1) Knowledge-based (2) Professional services	100 (52.4%) 175 (63.7%)	0.15 0.17	<0.05 <0.05	CS
		Eslamlou et al. (2021)	Not reported	Supervisor-report	Aviation	121 (60.3%)	0.17	<0.05	L
		He et al. (2018)	China	Supervisor-report	Various industries	335 (15.2%)	0.15	<0.05	CS
		Hu et al. (2022)	Pakistan	Self-report	Manufacturing	311 (34.7%)	0.20	<0.05	CS
		Hwang et al. (2023)	Korea	Self-report	Manufacturing	321 (50.2%)	−0.11	<0.05	CS
		Leung et al. (2014)	China	Supervisor-report	Education	189 (45.5%)	0.19	<0.01	CS
		Thundiyil et al. (2016)	China	Supervisor-report	Chemical	459 (46%)	−0.13	<0.05	CS
		Wang et al. (2023)	China	Supervisor-report	Manufacturing, finance and internet	1,384 (43.9%)	−0.09	<0.05	L
		Yang et al. (2022)	China	Supervisor-report	Banking	368 (79.4%)	−0.22	<0.05	L
	Educational level	Darvishmotevali et al. (2018)	Cyprus	Self-report	Hospitality	283 (52%)	0.13	<0.05	CS
		Gilmore et al. (2013)	China	Supervisor-report	Pharmaceutical	212 (74%)	0.36	<0.01	CS
		Liu et al. (2019)	China	Supervisor-report	High-tech	318 (24%)	0.14	< 0.05	L
		Man et al. (2020)	China	Supervisor-report	Medical	300 (78%)	0.12	<0.05	L
		Thundiyil et al. (2016)	China	Supervisor-report	Chemical	459 (46%)	0.18	<0.01	CS
		Tierney and Farmer (2011)	Not reported	Supervisor-report	Not reported	145 (68.8%)	0.24	<0.01	L
	Age	Hon et al. (2014)	China	Supervisor-report	High-tech, Manufacturing and Service	452 (47%)	−0.27	<0.05	CS
		Hu et al. (2019)	China	Self-report	Casual sector	275 (70.9%)	0.13	<0.05	CS
		Peng and Chen (2023)	Taiwan	Supervisor-report	Information technology	413 (75%)	0.12	<0.05	CS
		Thundiyil et al. (2016)	China	Supervisor-report	Chemical	459 (46%)	0.18	<0.01	CS
		Yang et al. (2022)	China	Supervisor-report	Banking	368 (79.4%)	−0.13	<0.05	L
	Job tenure	Hora et al. (2021a)	USA	Supervisor-report	Food manufacturing	335 (64%)	−0.10	<0.05	L
		Tierney and Farmer (2002)	Not reported	Supervisor-Report	(1) Consumer Goods (2) High-tech	502 (−%) 104 (−%)	0.20 —	<0.01 —	CS
		Yang et al. (2022)	China	Supervisor-report	Banking	368 (79.4%)	0.07	<0.05	L
	Industry type	Hu et al. (2022)	Pakistan	Self-report	Manufacturing	311 (34.7%)	0.24	<0.05	CS
	Marital status	Madjar et al. (2002)	Bulgaria	Supervisor-report	Apparel manufacturing	265 (97%)	0.13	<0.05	CS
	Use of digital platforms	Tonnessen et al. (2021)	Norway	Self-report	Not reported	237 (50%)	0.27	<0.01	CS
Personality	*Openness to Experience*	Herbst and Hausberg (2022)	Not reported	Self-report	Not reported	528 (−%)	0.45	<0.001	CS
		Shaw and Choi (2023)	USA	Supervisor-report	Consulting	170 (53.8%)	0.28	<0.001	CS
	Proactive personality	Chen et al. (2015)	Taiwan	Self-report	Manufacturing	337 (17%)	0.46	<0.01	CS
		Choi et al. (2021)	Korea	Self-report	Manufacturing and service	439 (30.3%)	0.74	<0.001	CS
		Li et al. (2020a)	Taiwan	Supervisor-report	Technology and manufacturing	125 (46.4%)	0.45	<0.01	L
		Sumaneeva et al. (2021)	Russia	Self-report	Hospitality	101 (65%)	0.44	<0.01	L
	Creative personality	Audenaert and Decramer (2018)	Belgium	Self-report	Industrial	213 (36%)	0.31	<0.01	CS
		Shalley et al. (2009)	USA	Self-report	Not reported	1,430 (51%)	0.16	<0.05	CS
	Autonomy orientation	Ye et al. (2014)	China	Supervisor-report	High-tech	120 (36.7%)	0.30	<0.01	CS
	Innovativeness	Chen et al. (2015)	Taiwan	Self-report	Manufacturing	337 (17%)	0.49	<0.01	CS
	Preference for creativity	Aleksic et al. (2016)	Slovenia	Self-report and supervisor-report	(1) Casual sector (2) Manufacturing	129 (59%) 165 (34%)	0.66 0.23	<0.01 <0.01	CS
	*Conscientiousness*	Shaw and Choi (2023)	USA	Supervisor-report	Consulting	170 (53.8%)	0.27	<0.001	CS
	Promotion focus	Liu et al. (2022)	China	Supervisor-report	Retail	206 (62.6%)	0.14	<0.05	CS
	*Extraversion*	Shaw and Choi (2023)	USA	Supervisor-report	Consulting	170 (53.8%)	0.27	<0.001	CS
	Optimism	Bouzari and Karatepe (2020)	Iran	Supervisor-report	Hospitality	187 (39.6%)	0.28	<0.01	L
	Neuroticism								
	Obsessive-compulsive personality	Abukhait et al. (2023)	UAE	Supervisor-report	Hospitality	252 (49.4%)	−0.28	<0.01	CS
	Resilience	Eslamlou et al. (2021)	Not reported	Supervisor-report	Aviation	121 (60.3%)	0.49	<0.01	L
	Resistance to Change	Hon et al. (2014)	China	Supervisor-report	High-tech, manufacturing and service	452 (47%)	−0.34	<0.01	CS
	Trait positive affectivity	Gilmore et al. (2013)	China	Supervisor-report	Pharmaceutical	212 (74%)	0.20	<0.01	CS
Cognitive ability	Cultural Intelligence	Hu et al. (2019)	China	Self-report	Casual sector	275 (70.9%)	−0.62	<0.01	CS
	Innovative cognitive style	De Stobbeleir et al. (2011)	Not reported	Supervisor-report	Consulting	456 (56%)	0.23	<0.01	CS
	Associative cognitive style	Shalley et al. (2009)	USA	Self-report	Not reported	1,430 (51%)	−0.23	<0.01	CS
	Perspective Taking	He et al. (2018)	China	Supervisor-report	Various industries	335 (15.2%)	0.31	<0.001	CS
Motivation	*Intrinsic Motivation*	Cheung and Zhang (2021)	China	Supervisor-report	(1) Knowledge-based (2) Professional services	100 (52.4%) 175 (63.7%)	0.24 0.53	<0.01 <0.01	CS
		Glaser et al. (2015)	Germany	Self-report	General population	830 (34.8%)	0.28	<0.01	CS
		Herbst and Hausberg (2022)	Not reported	Self-report	Not reported	528 (−%)	0.14	<0.05	CS
		Li et al. (2018)	China	Self-report	(1) Information technology (2) Information technology	141 (31%) 135 (46%)	0.24 0.41	<0.05 <0.01	CS
		Liu et al. (2019)	China	Supervisor-report	High-tech	318 (24%)	0.18	<0.01	L
		Makhija and Akbar (2019)	Pakistan	Supervisor-report	Information technology	240 (17.5%)	0.64	<0.05	CS
		Malik et al. (2015)	Pakistan	Supervisor-report	Not reported	181 (14%)	0.33	<0.01	CS
		Shaheen et al. (2020)	Pakistan	Supervisor-report	Banking	303 (33%)	0.51	<0.05	CS
		Shalley et al. (2009)	USA	Self-report	Not reported	1,430 (51%)	0.30	<0.01	CS
		Tonnessen et al. (2021)	Norway	Self-report	Not reported	237 (50%)	0.34	<0.001	CS
		Xie et al. (2020)	China	Supervisor-report	Not reported	386 (44.4%)	0.26	<0.001	CS
		Zhang and Bartol (2010)	China	Supervisor-report	Not reported	367 (34.8%)	0.66	<0.01	CS
	Challenge Intrinsic Motivation	Leung et al. (2014)	China	Supervisor-report	Education	189 (45.5%)	0.40	<0.01	CS
	Commitment to creativity	Yoon et al. (2015)	South Korea	Coworker-report	Not reported	241 (27%)	0.28	<0.01	CS
	Intrinsic rewards for creativity	Yoon et al. (2015)	South Korea	Coworker-report	Not reported	241 (27%)	0.21	<0.01	CS
	Extrinsic motivation								
	Extrinsic motivation	Herbst and Hausberg (2022)	Not reported	Self-report	Not reported	528 (−%)	0.56	<0.001	CS
	Importance of extrinsic rewards	Malik et al. (2015)	Pakistan	Supervisor-report	Not reported	181 (14%)	0.19	<0.05	CS
	Engagement								
	Creative process engagement	Herbst and Hausberg (2022)	Not reported	Self-report	Not reported	528 (−%)	0.37	<0.05	CS
		Khan and Abbas (2022)	Pakistan	Self-report	Service and manufacturing	303 (27.4%)	0.69	<0.01	CS
		Yang et al. (2021)	China	Supervisor-report	Electronics	347 (33.1%)	0.13	<0.05	L
		Zhang and Bartol (2010)	China	Supervisor-report	Not reported	367 (34.8%)	0.70	<0.01	CS
		Zheng et al. (2022)	China	Supervisor-report	Not reported	292 (47.3%)	0.33	<0.001	L
	Work engagement	Li et al. (2021)	China	Supervisor-report	High-tech	237 (39.7%)	0.39	<0.01	CS
		Peng and Chen (2023)	Taiwan	Supervisor-report	Information technology	413 (75%)	0.55	<0.01	CS
		Sumaneeva et al. (2021)	Russia	Self-report	Hospitality	101 (65%)	0.43	<0.01	L
	Green creative process engagement	Hu et al. (2022)	Pakistan	Self-report	Manufacturing	311 (34.7%)	0.35	<0.001	CS
	Motivation to goals								
	Goal self-concordance	Zhang et al. (2017)	China	Supervisor-report	Industrial	162 (41.4%)	0.29	<0.01	CS
	Learning goal	Liu et al. (2019)	China	Supervisor-report	High-tech	318 (24%)	0.18	<0.01	L
	Need for achievement	Hon (2012)	China	Self-report	Hospitality and service	219 (42%)	0.35	<0.01	CS
	Need satisfaction	Martinaityte et al. (2019)	Lithuania	Supervisor-report	Banking and cosmetics	329 (96%)	0.53	<0.01	CS
	Motivation (Others)								
	Green motivation	Hu et al. (2022)	Pakistan	Self-report	Manufacturing	311 (34.7%)	0.29	<0.05	CS
	Growth need strength	Shalley et al. (2009)	USA	Self-report	Not reported	1,430 (51%)	0.28	<0.01	CS
	Job embeddedness	Karatepe (2016)	Cameroon	Supervisor-report	Hospitality	212 (48%)	0.16	<0.05	CS
	Motivation (General)	Tonnessen et al. (2021)	Norway	Self-report	Not reported	237 (50%)	0.34	<0.001	CS
	Wisdom	Kalyar and Kalyar (2018)	Pakistan	Self-report	Service and manufacturing	753 (14.1%)	0.19	<0.01	CS
Emotions	Positive dimension								
	Positive affect	Gong and Zhang (2017)	China	Supervisor-report	Various industries	264 (49.2%)	0.41	<0.01	L
		Madjar et al. (2002)	Bulgaria	Supervisor-report	Apparel manufacturing	265 (97%)	0.20	<0.01	CS
		Qian and Jiang (2023)	China	Self-report	Various industries	107 (54.2%)	0.48	<0.01	L
		Thundiyil et al. (2016)	China	Supervisor-report	Chemical	459 (46%)	0.18	<0.01	CS
		Xie et al. (2020)	China	Supervisor-report	Not reported	386 (44.4%)	0.25	<0.001	CS
	Eudaimonic wellbeing	Villajos et al. (2019)	Spain	Self-report	Various industries	209 (60.8%)	0.40	<0.001	L
		Zhang and Zhao (2021)	China	Self-report	Not reported	288 (44.8%)	0.43	<0.01	CS
	Thriving at work	Bhatti et al. (2022)	Malaysia	Self-report	Information technology	227 (21.1%)	0.90	<0.05	CS
		Christensen-Salem et al. (2021)	Taiwan	Supervisor-report	Public sector	795 (34%)	0.49	<0.01	L
	Happiness	Khan and Abbas (2022)	Pakistan	Self-report	Service and manufacturing	303 (27.4%)	0.59	<0.01	CS
	Hedonic wellbeing	Zhang and Zhao (2021)	China	Self-report	Not reported	288 (44.8%)	0.59	<0.01	CS
	Negative dimension								
	Negative affect	Gong and Zhang (2017)	China	Supervisor-report	Various industries	264 (49.2%)	0.50	<0.01	L
	Stress	Kalyar and Kalyar (2018)	Pakistan	Self-report	Service and manufacturing	753 (14.1%)	−0.27	<0.01	CS
	Emotion regulation								
	Emotional intelligence	Cai et al. (2022)	Pakistan	Self-report	Education	237 (43.5%)	0.70	<0.05	CS
		Darvishmotevali et al. (2018)	Cyprus	Self-report	Hospitality	283 (52%)	0.43	<0.001	CS
Self-efficacy	Creative self-efficacy	Abdullah et al. (2017)	Pakistan	Self-report	Casual sector	322 (37%)	0.51	<0.05	CS
		Aleksic et al. (2016)	Slovenia	Self-report and supervisor-report	(1) Casual sector (2) Manufacturing	129 (59%) 165 (34%)	0.57 —	<0.01 —	CS
		Chiang et al. (2022)	Taiwan	Self-report	Education	341 (74.5%)	0.54	<0.01	L
		Choi et al. (2021)	Korea	Self-report	Manufacturing and service	439 (30.3%)	0.67	<0.001	CS
		Christensen-Salem et al. (2021)	Taiwan	Supervisor-report	Public sector	795 (34%)	0.44	<0.01	L
		Hora et al. (2021a)	USA	Supervisor-report	Food manufacturing	335 (64%)	0.12	<0.05	L
		Li et al. (2018)	China	Self-report	Information technology	135 (46%)	0.40	<0.01	CS
		Li et al. (2020b)	China	Supervisor-report	High-tech	346 (27.7%)	0.05	<0.05	L
		Liu et al. (2019)	China	Supervisor-report	High-tech	318 (24%)	0.31	<0.01	L
		Malik et al. (2015)	Pakistan	Supervisor-report	Not reported	181 (14%)	0.28	<0.01	CS
		Man et al. (2020)	China	Supervisor-report	Medical	300 (78%)	0.19	<0.01	L
		Nwanzu and Babalola (2022)	Nigeria	Self-report	Public and private sectors	148 (54.7%)	0.63	<0.01	CS
		Putra et al. (2023)	Indonesia	Self-report	Software development	253 (22%)	0.76	<0.05	CS
		Simmons et al. (2014)	USA	Supervisor-report	Not reported	128 (42%)	0.57	<0.01	CS
		Tang and Sun (2021)	China	Supervisor-report	Not reported	186 (52.7%)	0.22	<0.01	CS
		Thundiyil et al. (2016)	China	Supervisor-report	Chemical	459 (46%)	0.17	<0.01	CS
		Tierney and Farmer (2002)	Not reported	Supervisor-report	(1) Consumer Goods; (2) High-tech	502 (−%) 104 (−%)	0.17 0.24	<0.01 <0.01	CS
		Tierney and Farmer (2011)	Not reported	Supervisor-report	Not reported	145 (68.8%)	0.29	<0.01	L
		Torner (2023)	Colombia	Self-report	Electrical manufacturing	448 (39.1%)	0.58	<0.001	CS
		Wadei et al. (2021)	Ghana	Supervisor-report	Service industries	512 (38.7%)	0.74	<0.01	L
		Yulianti et al. (2022)	Indonesia	Self-report	Education	200 (61%)	0.37	<0.05	CS
		Yulianti and Usman (2019)	Indonesia	Self-report	Media	154 (−%)	0.32	<0.05	CS
	Self-efficacy	Ishaque et al. (2019)	Punjab	Supervisor-report	Education	302 (−%)	0.32	<0.05	CS
		Torner (2023)	Colombia	Self-report	Electrical manufacturing	448 (39.1%)	0.46	<0.001	CS
Performance	Organizational citizenship behavior	Al-Madadha et al. (2023)	Jordan	Self-report	Telecommunication	344 (55.9%)	0.77	<0.05	CS
		Chuang et al. (2019)	Taiwan	Coworker-report	Education	135 (81%)	0.53	<0.01	L
		He et al. (2018)	China	Supervisor-report	Various industries	335 (15.2%)	0.62	<0.001	CS
	Performance approach goal	Liu et al. (2019)	China	Supervisor-report	High-tech	318 (24%)	0.17	<0.01	L
	Task performance	Yang et al. (2022)	China	Supervisor-report	Banking	368 (79.4%)	0.78	<0.01	L
Others	Knowledge sharing	Ren et al. (2021)	China	Supervisor-report	Not reported	387 (51.2%)	0.34	<0.01	CS
		Wang et al. (2023)	China	Supervisor-report	Manufacturing, finance and internet	1,384 (43.9%)	0.41	<0.01	L
	Psychological capital	Gupta and Singh (2014)	India	Self-report	Public research	496 (25%)	0.69	<0.001	CS
		Ozturk and Karatepe (2019)	Russia	Supervisor-report	Hospitality	159 (67.9%)	0.28	<0.01	L
	Voice behavior	Hwang et al. (2023)	Korea	Self-report	Manufacturing	321 (50.2%)	0.77	<0.01	CS
		Song et al. (2017)	China	Self-report	Not reported	752 (53.7%)	0.47	<0.001	CS
	Creativity-oriented high-performance work systems	Martinaityte et al. (2019)	Lithuania	Supervisor-report	Banking and cosmetics	329 (96%)	0.57	<0.01	CS
	Diversity ideology	Cheung and Zhang (2021)	China	Supervisor-report	(1) Knowledge-based; (2) Professional services	100 (52.4%) 175 (63.7%)	0.23 0.25	<0.01 <0.01	CS
	Employee voice	Karkoulian et al. (2023)	Lebanon	Self-report	Banking	301 (46%)	0.76	<0.001	CS
	Environmental uncertainty	Darvishmotevali et al. (2018)	Cyprus	Self-report	Hospitality	283 (52%)	0.31	<0.001	CS
	Explicit knowledge contribution	Mohammed and Kamalanabhan (2019)	India	Self-report	Information technology	401 (31.2%)	0.42	<0.01	CS
	External digital knowledge sharing	Tonnessen et al. (2021)	Norway	Self-report	Not reported	237 (50%)	0.40	<0.001	CS
	Knowledge acquisition behavior	Sun et al. (2020)	China	Self-report	Not reported	365 (57.4%)	0.63	<0.01	CS
	Knowledge provision behavior	Sun et al. (2020)	China	Self-report	Not reported	365 (57.4%)	0.64	<0.01	CS
	Mindfulness	Khan and Abbas (2022)	Pakistan	Self-report	Service and manufacturing	303 (27.4%)	0.47	<0.01	CS
	Perceived organizational support	De Stobbeleir et al. (2011)	Not reported	Supervisor-report	Consulting	456 (56%)	0.16	<0.01	CS
	Prohibitive voice behavior	Prince and Kameshwar Rao (2022)	India	Self-report	Information technology	285 (71.2%)	0.39	<0.01	CS
	Promotive voice behavior	Prince and Kameshwar Rao (2022)	India	Self-report	Information technology	285 (71.2%)	0.47	<0.01	CS
	Tacit knowledge seeking	Mohammed and Kamalanabhan (2019)	India	Self-report	Information technology	401 (31.2%)	0.42	<0.01	CS
	Use of knowledge management systems	Herbst and Hausberg (2022)	Not reported	Self-report	Not reported	528 (−%)	0.62	<0.01	CS
	Voice toward peers	Li et al. (2022)	China	Supervisor-report	Manufacturing	206 (20.4%)	0.52	<0.01	L
	Work values: comfort	Ren et al. (2021)	China	Supervisor-report	Not reported	387 (51.2%)	−0.31	<0.01	CS
	Work values: competence	Ren et al. (2021)	China	Supervisor-report	Not reported	387 (51.2%)	0.26	<0.001	CS
	Work-life balance	Bouzari and Karatepe (2020)	Iran	Supervisor-report	Hospitality	187 (39.6%)	0.14	<0.05	L

Measurement indicates the type of assessment used for creative performance. *r* refers to the Pearson correlation coefficient.

The demographic factors found in the literature are gender, age, educational level, job tenure, industry type, marital status, and use of digital platforms. In terms of gender, the results were mixed, with five showing that women scored higher in creative performance on average than men (Aleksic et al., 2016; Cheung and Zhang, 2021; Eslamlou et al., 2021; Hu et al., 2022; Leung et al., 2014) while in five studies men obtained higher scores (He et al., 2018; Hwang et al., 2023; Thundiyil et al., 2016; Wang et al., 2023; Yang et al., 2022). Educational level appears to be positively associated with creative performance (Darvishmotevali et al., 2018; Gilmore et al., 2013; Liu et al., 2019; Man et al., 2020; Thundiyil et al., 2016; Tierney and Farmer, 2011). Time-related outcomes, such as age or job tenure, are inconclusive. Three studies reported that younger individuals scored higher on creative tasks (Hon et al., 2014; Thundiyil et al., 2016; Yang et al., 2022), whereas two studies found that older individuals obtained higher scores (Hu et al., 2019; Peng and Chen, 2023). Regarding job tenure, two studies reported a positive association between job tenure and creative performance scores (Tierney and Farmer, 2002; Yang et al., 2022). However, one study found that employees with less work experience scored higher (Hora et al., 2021a). Three other demographic factors have been examined in relation to creative performance: industry type, marital status, and use of digital platforms. Hu et al. (2022) observed differences by industry type, as employees in the manufacturing sector were more motivated by innovative ideas and solutions than employees in the service sector. Married individuals demonstrated greater creativity than their unmarried counterparts (Madjar et al., 2002). The use of digital platforms (e.g., tools for video meetings, enterprise social media, file-sharing, etc.) positively correlated with creative performance (Tonnessen et al., 2021).

Personality-related factors associated with creative performance were classified according to the Big Five, a model that describes five broad traits of personality—openness, conscientiousness, extraversion, agreeableness, and neuroticism—that are widely accepted in the workplace (Salgado and De Fruyt, 2017). Among these traits, openness shows the strongest empirical support (Herbst and Hausberg, 2022; Shaw and Choi, 2023). Several personality facets also show consistent positive associations with creative performance, including creative personality (Audenaert and Decramer, 2018; Shalley et al., 2009), autonomy orientation (Ye et al., 2014), innovativeness (Chen et al., 2015), and preference for creativity (Aleksic et al., 2016), with a special emphasis on the proactive personality (Chen et al., 2015; Choi et al., 2021; Li et al., 2020a; Sumaneeva et al., 2021). Associations for facets related to conscientiousness (Liu et al., 2022; Shaw and Choi, 2023) and extraversion (Bouzari and Karatepe, 2020; Shaw and Choi, 2023) align with findings from other performance research, with both traits showing positive relationships with creative performance. In fact, openness, conscientiousness and extraversion were the only personality constructs with empirical evidence supporting their association with creative performance at the trait level. Neuroticism is characterized by the tendency to experience unfavorable emotions. Constructs that can be comprised within neuroticism, like obsessive-compulsive personality (Abukhait et al., 2023) and resistance to change (Hon et al., 2014), show negative associations, as expected from the literature. Consequently, traits such as resilience (Eslamlou et al., 2021), which opposes neuroticism, and trait positive affectivity (Gilmore et al., 2013) are positively associated with creative performance. Agreeableness is the only trait that does not show associations in terms of creative performance.

Four personal factors were included in cognitive ability. Hu et al. (2019) observed that cultural intelligence is positively associated with employees’ creative performance. Innovative cognitive style shows a positively association with creative performance (De Stobbeleir et al., 2011). Similarly, perspective-taking is positively related to creative performance (He et al., 2018). However, an associative cognitive style is associated with lower creative performance, a pattern that may reflect its reliance on existing connections, which could relate to lower levels of novelty in idea generation (Shalley et al., 2009).

Evidence shows that motivation is consistently associated with creative performance. The constructs have been categorized as intrinsic motivation, extrinsic motivation, engagement, and others. Concerning intrinsic motivation, intrinsic motivation itself (Cheung and Zhang, 2021; Glaser et al., 2015; Herbst and Hausberg, 2022; Li et al., 2018; Liu et al., 2019; Makhija and Akbar, 2019; Malik et al., 2015; Shaheen et al., 2020; Shalley et al., 2009; Tonnessen et al., 2021; Xie et al., 2020; Zhang and Bartol, 2010), challenging intrinsic motivation (Leung et al., 2014), commitment to creativity (Yoon et al., 2015), and intrinsic rewards for creativity (Yoon et al., 2015) show positive associations with creative performance. Regarding extrinsic motivation, constructs such as extrinsic motivation itself (Herbst and Hausberg, 2022) and the importance of extrinsic rewards (Malik et al., 2015) are positively related to creative performance. Another motivational construct related to creative performance is engagement, defined as employees’ commitment to their organizational roles (Reig-Botella et al., 2024). The results show that creative process engagement (Herbst and Hausberg, 2022; Hu et al., 2022; Khan and Abbas, 2022; Yang et al., 2021; Zhang and Bartol, 2010; Zheng et al., 2022) and work engagement (Li et al., 2021; Peng and Chen, 2023; Sumaneeva et al., 2021) are positively associated with creative performance. Similarly, motivation for goals is positively associated with creative performance (Hon, 2012; Liu et al., 2019; Martinaityte et al., 2019; Zhang et al., 2017). Other more specific constructs are also associated with creative performance, such as green motivation (Hu et al., 2022), growth need strength (Shalley et al., 2009), job embeddedness (Karatepe, 2016), general motivation (Tonnessen et al., 2021) or wisdom (Kalyar and Kalyar, 2018).

Personal constructs related to emotions have been classified into three categories: positive dimension, negative dimension, and emotion regulation. Within the positive dimension, constructs such as positive affect (Gong and Zhang, 2017; Madjar et al., 2002; Qian and Jiang, 2023; Thundiyil et al., 2016; Xie et al., 2020), eudaimonic well-being (Villajos et al., 2019; Zhang and Zhao, 2021), thriving at work (Bhatti et al., 2022; Christensen-Salem et al., 2021) and happiness (Khan and Abbas, 2022), hedonic well-being (Zhang and Zhao, 2021), had a positive association with creative performance. However, in the negative dimension, negative affect (Gong and Zhang, 2017) and stress (Kalyar and Kalyar, 2018) are negatively related to creative performance. In addition, within the emotion regulation category, emotional intelligence (Cai et al., 2022; Darvishmotevali et al., 2018) is positively associated with creative performance.

Self-efficacy shows a positive relationship with creative performance (Ishaque et al., 2019; Torner, 2023). However, most studies report that creative self-efficacy is positively associated with creative performance (Abdullah et al., 2017; Aleksic et al., 2016; Chiang et al., 2022; Choi et al., 2021; Christensen-Salem et al., 2021; Hora et al., 2021a; Li et al., 2018; Li et al., 2020b; Liu et al., 2019; Malik et al., 2015; Man et al., 2020; Nwanzu and Babalola, 2022; Putra et al., 2023; Simmons et al., 2014; Tang and Sun, 2021; Thundiyil et al., 2016; Tierney and Farmer, 2002, 2011; Torner, 2023; Wadei et al., 2021; Yulianti et al., 2022; Yulianti and Usman, 2019).

Studies also investigate the relationship between creative performance and other dimensions of job performance. As expected, there is a positive relationship between creative performance and the dimensions of organizational citizenship behavior (Al-Madadha et al., 2023; Chuang et al., 2019; He et al., 2018) and task performance (Yang et al., 2022). Similarly, performance-approach goal orientation is positively related to creative performance (Liu et al., 2019). As part of the broader job performance construct, these findings should not be interpreted as evidence of a causal relationship, but rather as indicating covariation among performance dimensions.

A total of 23 personal factors were grouped under the “others” category (e.g., knowledge sharing; psychological capital). These factors are conceptually heterogeneous and do not align within the broader categories that were developed inductively after examining the set of reviewed studies. Because of their specificity and limited recurrence across the literature, a detailed discussion of each factor is beyond the scope of this review. Readers interested in any of these variables may consult the corresponding primary studies for a fuller examination.

**Figure 3:**
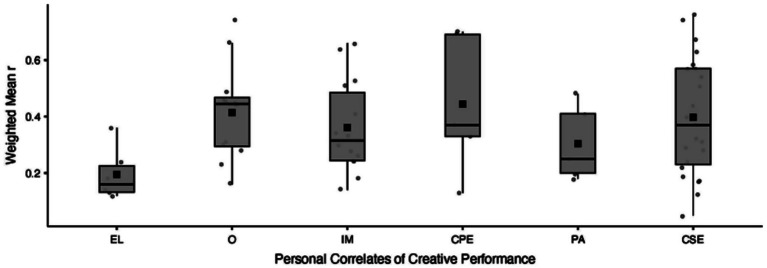
Key factors on creative performance. *Note*. EL: Educational Level; O: Openness; IM: Intrinsic Motivation; CPE: Creative Process Engagement; PA: Positive Affect; CSE: Creative Self-Efficacy.

Finally, we aimed to identify which personal factors are most consistently associated with creative performance in the literature. Given the distribution of the papers reporting this relationship (*M* = 1.96, *SD* = 2.87), we focused on constructs with five or more studies (+1 *SD*) examining their association with creative performance. On this basis, six personal factors were retained. Weighted arithmetic means showed a small but significant positive association for educational level (*r_w_* = 0.19, 95% *CI* [0.12, 0.27], *p* < 0.001; *I*^2^ = 55.66%), and moderate to strong positive associations for openness (*r_w_* = 0.46, 95% *CI* [0.33, 0.59], *p* < 0.001; *I*^2^ = 93.76%), intrinsic motivation (*r_w_* = 0.39, 95% *CI* [0.28, 0.50], *p* < 0.001; *I*^2^ = 93.37%), creative process engagement (*r_w_* = 0.52, 95% *CI* [0.23, 0.80], *p* < 0.001; *I*^2^ = 97.39%), positive affect (*r_w_* = 0.31, 95% *CI* [0.18, 0.43], *p* < 0.001; *I*^2^ = 81.53%), and creative self-efficacy (*r_w_* = 0.45, 95% *CI* [0.34, 0.56], *p* < 0.001; *I*^2^ = 95.30%). Given the very high heterogeneity observed for most constructs, these weighted mean correlations should be interpreted with caution and regarded primarily as summary indicators of the central tendency of the reported associations. As illustrated in Figure 3, the variability of effect sizes across studies is substantial, underscoring the need to consider contextual and methodological differences when interpreting these findings.

## 6 Discussion

The present paper aims to identify the main personal correlates of creative performance. For this purpose, we performed a systematic review of the literature. The personal factors identified across studies show substantial heterogeneity in both conceptual focus and empirical findings. In addition to the direct associations summarized in this section, the review also identified a substantial number of indirect effects (39 moderations and 18 mediations) reflecting the complex interplay among personal factors and their links to creative performance. Given the heterogeneity and volume of these results, they are presented in detail in the Supplementary material. Nevertheless, our results suggest that six personal factors are most consistently associated with creative performance. We will now present the implications of these results.

The weighted arithmetic means revealed substantial heterogeneity for most personal factors (I^2^ ranging from 56 to 97%), indicating that the strength of these associations varies markedly across studies. This variability can be explained by differences in how creative performance was measured (self-reports, supervisor-report, or coworker-report), the diversity of industries represented, and cultural contexts that shape how creativity-related constructs operate. Together, these factors suggest that creative performance is highly context-dependent, and that personal correlates should be interpreted with caution and in relation to the settings in which they were examined.

First, education has been consistently associated with higher levels of creative performance by providing individuals with new knowledge, skills, and perspectives that can expand their creativity and help them generate innovative ideas (Amabile, 1996; Liu et al., 2019). Moreover, it can teach individuals how to think critically and develop problem-solving skills that are essential components of creativity. Specifically, a higher educational level was associated with a slightly higher level of creative performance (Tierney and Farmer, 2011). This may be because people with higher educational levels have more knowledge and skills to draw from when engaging in creative work.

Second, openness was identified as the most relevant Big Five dimension associated with creative performance. Individuals who score high on openness tend to be more imaginative and open-minded, which can lead to increased creativity in their work (George and Zhou, 2001). This result is interesting compared with other performance dimensions such as contextual performance or adaptative performance, where conscientiousness and agreeableness tend to display higher associations than openness (Ramos-Villagrasa et al., 2022).

Third, intrinsic motivation is consistently associated with higher levels of creative performance because individuals driven by an internal need to provide meaning to their work find the task inherently enjoyable and challenging (Amabile, 1997). When intrinsically motivated, employees tend to question conventional work methods, exhibit a greater desire to acquire knowledge, and think innovatively about new work processes (Dewett, 2007).

Similarly, the review also underscores the influence of creative process engagement (CPE) as a motivational factor. CPE is the cognitive process employees engage in to produce creative outcomes (Amabile and Pillemer, 2012). Zhang and Bartol (2010) propose that CPE fosters creativity by enabling employees to more effectively define problems, integrate information, and explore new ideas. Following Yang et al. (2021), individuals who display CPE tend to develop a more precise understanding of the issue at hand, which, in turn, allows them to offer more useful and distinctive solutions. Second, when employees seek information from a variety of sources, they can reassess the status quo with new perspectives and develop novel ideas by integrating this new information with their existing knowledge. Thirdly, the generation of new ideas through exploration facilitates the emergence of originality, as creativity frequently emerges from the generation of a multitude of alternatives through the interconnection of disparate sources of information. Highly engaged employees tend to exhibit high levels of energy and enthusiasm, are deeply involved in their work, and feel strongly connected to their tasks (Bakker and Demerouti, 2014).

Five, concerning positive affect, it is a part of dispositional affectivity and characterizes excited, joyful, enthusiastic, and exhilarate people (Cropanzano et al., 1993). Individuals with high positive affect typically adopt a broad cognitive processing style (Thundiyil et al., 2016). The efficacy of strategies for enhancing creative performance may vary depending on how individuals regulate their affect (Gong and Zhang, 2017).

Lastly, creative self-efficacy (CSE) reflects an individual’s belief in their ability to successfully engage in creative tasks, revealing their confidence in generating new ideas, finding creative solutions, and performing creatively within a specific domain (Hu et al., 2018; Nwanzu and Babalola, 2022). This result is expected because self-efficacy should be studied in relation to specific domains of functioning and particular contexts (Bandura, 1997). CSE, as an individual’s self-confidence in the creation of novel products, is a specific facet of self-efficacy (Tierney and Farmer, 2002), namely one’s perceived ability to succeed in specific tasks (Bandura, 1997). These findings indicate that conceptualizing CSE as a foundation for creative endeavors is paramount. CSE enables individuals to discern opportunities within challenges and persevere in adversity (Tierney and Farmer, 2011). When professionals demonstrate a profound mastery of their field of expertise and the requisite skills, they exhibit a higher CSE, enabling them to conceptualize innovative solutions (Capron-Puozzo and Audrin, 2021). However, it is important to note that many studies examining the association between CSE and creative performance rely on self-report measures for both constructs. This measurement approach may inflate the observed correlations due to common method variance, particularly when creativity ratings are collected from the same source as CSE. Therefore, although the association between CSE and creative performance is robust, it should be interpreted with caution, and future research would benefit from incorporating multi-source or multi-method assessments to reduce potential bias.

### 6.1 Research implications

This study makes two theoretical contributions to the literature. First, the findings of this research have allowed us to identify the personal factors most consistently associated with creative performance. This contribution helps to clarify the nomological network of creative performance and its integration within the multidimensional job performance perspective. We wanted to focus on personality and cognitive ability. These constructs are among the personal factors most consistently associated with job performance (Sackett et al., 2022), but the present review highlights some differences between creative performance and other performance dimensions. Concerning personality, the most relevant trait for creative performance is openness, not conscientiousness or neuroticism. However, more research is needed to consider the remaining traits (Shaw and Choi, 2023). Cognitive ability does not emerge as one of the primary personal factors associated with creative performance, largely because its role has been examined only in a limited number of studies. Nevertheless, individuals with higher cognitive ability typically learn faster, solve problems more effectively, and adapt better to new situations (Sternberg and Sternberg, 2012). This is especially relevant in jobs that require problem-solving, decision-making, and continuous learning skills (Shalley et al., 2009).

Secondly, our review revealed some gaps in the existing research, suggesting a need for further investigation. More research is needed to delve into sociodemographic variables, as the findings regarding gender, age, and job tenure are inconclusive or mixed. Regarding gender, according to the meta-analysis conducted by (Hora et al., 2021b), evidence indicates a minor yet statistically significant gender difference in creative performance, with men slightly outperforming women. The study attributes this disparity to societal factors, including gender roles and cultural variables. However, it also suggests that initiatives to promote equality could diminish or eradicate this gap in creative performance. (Hora et al., 2021a) find that CSE is a potential explanatory mechanism for the peculiar association between gender and creative performance. To some extent, the observed gender gap may be associated with women’s lower self-confidence in their capacity for creative performance. The study indicates that, despite evolving societal stereotypes and changing working conditions, women may still encounter gender-based biases that can diminish their confidence in their creative abilities and restrict their creative performance. Moreover, further research is required to determine the influence of age and job tenure on creative performance. In clarifying how these variables influence creativity, valuable insights can be gained into how employees’ experiences and life stages affect their capacity to generate innovative ideas and solutions. Further investigation in this domain will assist organizations in developing strategies to enhance creative performance across diverse age groups and career stages.

### 6.2 Practical implications

In organizational settings, creative performance emerges from the combined influence of individual characteristics, the work environment, and the dynamic interaction between both. From a practical perspective, identifying the personal factors associated with creative performance and understanding which are the most consistently in the literature can guide human resource practices that influence organizational performance (Villajos et al., 2019). One relevant consideration for practitioners is the (relative) stability or variability of the personal factors associated with creative performance over time. Positive affect, personality traits, and intrinsic motivation are relatively stable attributes. In contrast, educational level, creative self-efficacy, and creative engagement may be more amenable to change, depending on the situation or intervention. This information can guide human resource practices by indicating which personal factors may be more relevant to consider in different process.

One of these processes is personnel selection. Suppose we are hiring people who need to display creative performance in their jobs. In that case, we might consider applicants with a high level of education and a strong openness to new experiences.

Another process is training and development. Training programs can immerse employees in diverse scenarios involving critical aspects of creative tasks, thereby providing opportunities to enhance their creative performance. This aligns with Bandura (1997) four sources of self-efficacy. First, mastery experiences can be fostered by providing employees with opportunities to successfully complete challenging creative tasks, thereby strengthening their belief in their creative abilities. Second, vicarious experiences can be cultivated by exposing employees to role models or peers who demonstrate creative success, allowing them to learn and gain confidence through observation. Third, social persuasion involves providing employees with encouragement and positive feedback that reinforces their creative potential. And four, addressing physiological states means creating an environment that reduces stress and anxiety, helping employees feel more capable and confident about their creative behaviors.

Furthermore, training programs could also increase employees’ engagement in the creative process by immersing them in diverse, challenging scenarios that stimulate creative behaviors. This training may be based on three basic dimensions: (i) identifying a problem; (ii) processing information; and (iii) generating an idea to solve the identified problem (Zhang and Bartol, 2010). By engaging in structured activities and real-world problem-solving tasks, employees are encouraged to actively participate in each stage of the creative process, from initial brainstorming to final implementation. Training programs to foster positive affect could enhance creative performance by boosting employees’ mood and emotional state. Positive affect has been shown to enhance employees’ willingness to take risks and explore innovative ideas, which, in turn, can lead to improved creative output (Qian and Jiang, 2023). Career management is related to training and development, where trying to guide workers into positions that match their intrinsic motivation can lead to improved creative performance.

The last process where we can outline the implications of our review is performance appraisal systems. Given that some personal factors are consistently associated with creative performance and that not all are stable over time, differences in employees’ creative performance may emerge and should therefore be examined periodically.

### 6.3 Limitations and further research

As with any study, the present research has some limitations that should be acknowledged. Firstly, although we considered the Web of Science and Scopus databases, which are well-known for their comprehensive research coverage, we may have unintentionally excluded relevant studies not indexed within these platforms. Future research could gain from expanding the inclusion criteria or using other databases to reduce the likelihood of missing important information. Secondly, the systematic literature review used publication as an inclusion criterion, a common practice that may introduce publication bias. Because only published studies were included, studies with non-significant or weaker associations may be underrepresented, which could lead to an overestimation of the magnitude of the reported relationships. Thirdly, the current study’s methodology is limited to a systematic literature review. This method is useful for identifying trends and themes across studies, but future research may employ meta-analytic techniques to extend our results. Additionally, as our research is focused only on quantitative research, further research should also consider qualitative studies to improve the scope of the present review. Moreover, because many of the studies included in this review relied on cross-sectional designs, the direction of the observed associations cannot be established, and reverse causality remains possible.

Our review also leads to proposing some ideas for further research. First, we believe that a systematic review focused on the contextual factors associated with creative performance could complement the present research. In addition, an analysis of the measures of creative performance, their functioning, and their limitations might help advance future research on the construct. We also suggest that examining creative performance jointly with the remaining dimensions of job performance would lead to better insights about what each dimension has (and does not have) in common with the others. Furthermore, the systemic nature of creativity imposes important constraints on an individual-centered approach. The effects of personal characteristics on creative performance may be strengthened, attenuated, or obscured by contextual conditions such as team dynamics, leadership, organizational climate, or task structure (Amabile and Pillemer, 2012). In real organizational settings, it is therefore essential to consider these additional influences, as focusing solely on individual factors may underestimate the extent to which person–context interactions shape creative behavior.
