# Supplementary material for: Personal correlates of creative performance: A systematic review
Source: Front Psychol. 2026 Jan 30;16:1727094. doi: 10.3389/fpsyg.2025.1727094 (PMC12900707; doi:10.3389/fpsyg.2025.1727094)
Supplement: Supplementary file 1 [file Supplementary_file_1.docx]

Supplementary Material: Personal factors with indirect effects on predicting creative performance

The present study examines the personal factors that contribute to creative performance. We evaluated the direct (n = 79) and indirect effects (n = 57) of the relationships between variables, with an emphasis on mediation (n = 18; 31.6%) and moderation (n = 39; 68.4%) as forms of indirect influence. Specifically, our analysis was limited to relationships where the antecedent was a personal factor. This approach allowed us to identify the complex pathways through which personal attributes exert their influence, emphasizing the significant impact of personal factors in shaping both direct and indirect effects in a range of scenarios. Information regarding the indirect effects is presented in Table 1. The relationship between two variables can be direct or indirect. A direct relationship exists between two variables when one variable influences the other without the involvement of an intermediary variable. An indirect relationship occurs when a third variable affects both variables being studied and mediates the relationship between them. Moderation is another concept that differs from mediation, in that the relationship between two variables is influenced by the presence of a third variable. In this case, the third variable acts as a moderator. The following section presents the indirect effects of personal factors on creative performance, focusing on the mediation and moderation processes.

# Demographic factors

Only one indirect effect was identified for the demographic factors. Yang et al. (2022) found that job tenure negatively moderates the indirect relationship between education and creative performance via task performance. Specifically, this positive indirect association becomes weaker among employees with higher job tenure compared to those with lower job tenure.

# Personality factors

Personal factors that are related to personality and have an indirect effect include autonomy orientation, innovativeness, obsessive-compulsive personality, preference for creativity, proactive personality, resistance to change, and trait positive affectivity.

Regarding autonomy orientation, Ye et al. (2014) suggest that well-being plays a mediating role in the relationship between autonomy orientation and creative performance.

Following with innovativeness, Chen et al. (2015) found that the relationship between innovativeness and external bridging networks had a negative impact on creative performance. However, the relationship between innovativeness and internal bridging networks may influence the effect of innovativeness on creative performance. Specifically, individuals with high levels of internal bridging networks may experience a stronger positive effect of innovation on creative performance.

Abukhait et al. (2023) found that the relationship between an individual's obsessive-compulsive personality and a manager's behavior can impact an employee's capacity to demonstrate creative performance in the workplace. Consequently, the efficacy of coaching managers may fluctuate to mitigate the adverse effects of employees' personalities on their performance.

Two potential factors may contribute to the preference for creativity. Aleksic et al. (2016) examined the relationship between preference for creativity and creative output in the presence of clear outcome goals. This study aims to determine whether work enjoyment mediates this relationship. The results indicated that the manipulation of clear goals led to individuals' preferences for creativity, resulting in higher work enjoyment and, in turn, superior ratings of their creative performance. In addition, the results indicate that the clarity of outcome goals plays a moderating role in the association between creativity preference and creative behavior at work. This implies that clear outcome goals are necessary for creative preference to manifest in creative performance.

Proactive personality is the personal factor that exhibits the most indirect effects. In a study conducted by Choi et al. (2021), it was determined that creative self-efficacy plays a pivotal role in linking proactive personality to creative performance. This is because it strengthens employees’ beliefs and confidence in their creative behaviors. In addition, the authors identified another effect. Psychological safety can positively impact the connection between proactive personality and creative performance. This positive interaction between proactive personality and psychological safety may provide employees with important resources to enhance their creative performance by boosting their confidence, motivation, and trust in organizational rewards and support. Li et al. (2020) demonstrated that job crafting partially mediates the relationship between proactive personality and creative performance. Furthermore, this study identified evidence of moderated mediation, whereby the indirect effect of proactive personality on creative performance via job crafting is moderated by high-involvement work systems (HIWS). Specifically, when the HIWS was low, proactive personality exhibited a notable indirect effect on creative performance through job crafting. However, when the HIWS was high, an indirect effect was not observed. Sumaneeva et al. (2021) found that work engagement functions as a partial mediator in the relationships between proactive personality and creative performance. This suggests that, while there is a direct correlation between proactive personality and creative performance, the relationship is also partially explained by employees' level of work engagement. Following Chen et al., (2015) the relationship between proactiveness and upper management networks demonstrates that the influence of proactiveness on creative performance can vary according to the degree of connection to upper management. The study revealed that proactivity has a greater impact on creative performance when the level of connection to upper management is high than when it is low.

Regarding resistance to change, Hon et al. (2014) showed that the negative relationship between resistance to change and employees' creative performance can be moderated by supportive coworkers, organizational modernity, and empowering leadership. These factors can mitigate the negative impact of the resistance to change on creative performance.

Finally, Gilmore et al. (2013) revealed that the level of positive affectivity among followers influences the relationship between transformational leadership and creative performance. Transformational leadership was positively related to follower creative performance among followers who exhibited lower levels of trait positive affectivity. Conversely, transformational leadership was not significantly related to follower creative performance among followers who demonstrated higher levels of positive affectivity.

# Cognitive ability

In the cognitive ability category, two moderation effects were found in the construct of cultural intelligence, and one mediation effect was identified in the innovative cognitive style. As posited by Hu et al. (2019), two moderating effects are observed between cultural intelligence and conflict, namely, relationship conflict and task conflict. Relationship conflict positively moderates the relationship between cultural intelligence and creative performance, while task conflict negatively moderates this relationship. According to De Stobbeleir et al. (2011), the relationship between cognitive style and perceived organizational support for creativity and creative performance is partially mediated by feedback inquiry. The results indicate that employees who frequently seek feedback are more likely to exhibit creative performance. This is because cognitive style and perceived organizational support for creativity influence the frequency of feedback inquiry, which consequently boosts creative performance.

# Motivation

Personal factors categorized under motivation that exhibit indirect effects include creative process engagement, extrinsic rewards, green motivation, growth need strength, and intrinsic motivation. Zheng et al. (2022) found that the interaction between creative process engagement and the climate for innovation significantly impacts creative performance, with higher innovation climates amplifying the positive effect of creative process engagement. Malik et al. (2015) found that extrinsic rewards moderate the relationship between the importance of extrinsic rewards and creative performance. Extrinsic rewards significantly enhance the creative performance of employees who consider them important. Conversely, the absence of such rewards has been shown to exert a detrimental influence on the creative performance of employees who do not prioritize these forms of compensation. The study also revealed that creative self-efficacy exerts a significant moderating influence on the relationship between extrinsic rewards and creative performance. For employees with high creative self-efficacy, the relationship was positive, whereas it was negative for those with low creative self-efficacy. In terms of a specific construct of motivation, Hu et al. (2022) examined the mediating effect of green creative process engagement (GCPE) on the relationship between employees' green motivation (GM) and green creative performance (GCP). The results showed that GCPE partially mediated the relationship between GM and GCP, indicating that employee engagement in green creative processes played a role in translating green motivation into actual green creative performance in the workplace. Regarding growth need strength, Shalley et al. (2009) examined how job complexity and the work context impact the relationship between growth need strength and creative performance. This reveals that a supportive work context strengthens this relationship, whereas job complexity weakens it. Finally, Cheung and Zhang (2021) suggested that employees with strong rather than weak personal factors, such as intrinsic motivation, benefit more from these office design dimensions (i.e., design for autonomy and interactive office design) in terms of having higher levels of creativity.

# Emotions

The factors categorized under emotions were emotional intelligence, hedonic well-being, negative affect, and positive affect, with the latter having the highest number of indirect effects. Darvishmotevali et al. (2018) found the moderating effect of cultural intelligence on the relationship between emotional intelligence and creative performance. Specifically, this connection was stronger for individuals with high cultural intelligence than for those with low cultural intelligence. Employees with high cultural intelligence can quickly adapt to different cultural environments and develop innovative approaches to improve customer satisfaction, whereas emotional intelligence enables them to understand and manage emotions effectively. Regarding hedonic well-being, Zhang and Zhao (2021) show that when direct and instrumental achieving styles are rated more favorably, the effect of hedonic well-being on creative performance is stronger. Chuang et al. (2019) suggest that employees who engage in organizational citizenship behavior (OCB) have the capacity to transform negative affect into creative performance. The findings related by Thundiyil et al. (2016) indicate that creative self-efficacy positively impacts creative performance in scenarios with low positive affect. The negative impact of negative affect on performance weakened with higher self-efficacy. The authors also showed that creative self-efficacy enhances creative performance when they experience both low and high levels of positive and negative affect, respectively. Gong and Zhang (2017) indicate that the direct effect of positive affect on creative performance is moderated by negative affect. Specifically, the relationship between positive affect and creative performance was stronger for individuals with higher levels of negative affect than for those with lower negative affect. Additionally, the direct impact of positive affect change on creative performance is moderated by negative affect change. The slope of the relationship between positive affect increase and creative performance was relatively stronger for negative affect decrease than for negative affect increase. According to Qian and Jiang (2023) perceived organizational support (POS) enhances the positive effects of positive emotions on employees’ creative performance. The correlation between positive emotions and employees’ creative performance was strong and positive when POS was high; this relationship was weaker when POS was low.

# Self-Efficacy

Several constructs have been found to have indirect effects on both job self-efficacy and creative self-efficacy. Christensen-Salem et al. (2021) found that thriving at work partially mediated the relationship between creative self-efficacy and creative performance. Employees with higher levels of thriving at work demonstrated a more favorable relationship between creative self-efficacy and creative performance. The results also showed that perceived work significance and task interdependence independently and jointly moderated the relationship between creative self-efficacy and creative performance, such that the relationship was more pronounced when both perceived work significance and task interdependence were high rather than low. Nwanzu and Babalola (2022) found that creative self-efficacy moderates the impact of psychological ownership on creative performance. Thus, the positive influence of psychological ownership on creative performance was stronger when creative self-efficacy was higher than when it was lower. Tierney & Farmer (2002) developed a new efficacy construct specific to job creativity by integrating the research on self-efficacy and creativity. Their study suggested that the level of job self-efficacy influences the relationship between creative self-efficacy and creative performance only when employees strongly believe in their creative abilities. However, job self-efficacy does not have a moderating influence on employees with low CSE. According to Ishaque et al. (2019), two partial mediations exist between self-efficacy and creative performance. Thus, on the one hand, the challenge of rewarding creativity positively mediates the relationship between self-efficacy and creative performance. However, self-efficacy has a negative relationship with creative performance through threat assessment of rewards for creativity.

# “Others” category

In the final category, labeled "others," seven mediating effects and four moderating effects were identified. These factors do not fall into the categories above. As a result, we will not provide a detailed analysis of each factor in this report. For readers who are interested in a particular determinant, we recommend referring to the study in which it has been analyzed in greater detail.

Table 1. Personal Factors with Indirect Effects on Predicting Creative Performance

| **Category** | **Construct** | **R** | **Reference** |
| --- | --- | --- | --- |
| Demographic | Educational Level – Task Performance * Job Tenure – CP | - | Yang et al. (2022). |
| Personality | Autonomy orientation – Well-being – CP | + | Ye et al. (2014). |
|  | Innovativeness * External Bridging Networks | - | Chen et al. (2015)**.** |
|  | Innovativeness * Internal Bonding Networks | + | Chen et al. (2015)**.** |
|  | Obsessive–Compulsive Personality * Manager Coaching Behavior | + | Abukhait et al. (2023)**.** |
|  | Preference For Creativity * Clear Outcome Goals | + | Aleksic et al. (2016)**.** |
|  | Preference for creativity – Work enjoyment – CP | + | Aleksic et al. (2016). |
|  | Proactive Personality – Creative Self-Efficacy – CP | + | Choi et al. (2021). |
|  | Proactive Personality * Psychological Safety | + | Choi et al. (2021)**.** |
|  | Proactive Personality – Job Crafting – CP | + | Li et al. (2020). |
|  | Proactive Personality * High-involvement Work Systems – Job Crafting – CP | - | Li et al. (2020)**.** |
|  | Proactive Personality – Work Engagement – CP | + | Sumaneeva et al. (2021). |
|  | Proactive Personality * Upper Management Networks | + | Chen et al. (2015)**.** |
|  | Resistance to change * Coworkers | + | Hon et al. (2014)**.** |
|  | Resistance to change * Leadership | + | Hon et al. (2014)**.** |
|  | Resistance to change * Modernity | + | Hon et al. (2014)**.** |
|  | Trait Positive Affectivity * Transformational Leadership | - | Gilmore et al. (2013)**.** |
| Motivation | Creative Process Engagement * Climate for Innovation | + | Zheng et al. (2022)**.** |
|  | Extrinsic Rewards * Importance of Extrinsic Rewards | + | Malik et al. (2015)**.** |
|  | Extrinsic Rewards * Creative Self Efficacy | + | Malik et al. (2015)**.** |
|  | Green Motivation – Green Creative Process Engagement – CP | + | Hu et al. (2022). |
|  | Growth Need Strength * Job Complexity | - | Shalley et al. (2009)**.** |
|  | Growth Need Strength * Supportive Work Context | + | Shalley et al. (2009)**.** |
|  | Growth Need Strength * Supportive Work Context * Job Complexity | - | Shalley et al. (2009)**.** |
|  | Intrinsic Motivation * Design for Autonomy | + | Cheung & Zhang (2021). |
|  | Intrinsic Motivation * Interactive Office Design | + | Cheung & Zhang (2021). |
| Self-Efficacy | Creative Self-Efficacy – Thriving at Work – CP | + | Christensen-Salem et al. (2021)**.** |
|  | Creative Self-Efficacy * Work Significance * Task Interdependence | + | Christensen-Salem et al. (2021)**.** |
|  | Creative Self-Efficacy * Work Significance | + | Christensen-Salem et al. (2021)**.** |
|  | Creative Self-Efficacy * Task Interdependence | + | Christensen-Salem et al. (2021)**.** |
|  | Creative Self Efficacy * Psychological Ownership | + | Nwanzu & Babalola (2022). |
|  | Creative Self-Efficacy * Job Self-Efficacy | + | Tierney & Farmer (2002). |
|  | Self-Efficacy – Challenge Appraisal of Reward for Creativity – CP | + | Ishaque et al. (2019). |
|  | Self-Efficacy – Threat Appraisal of Reward for Creativity – CP | - | Ishaque et al. (2019). |
| Emotions | Emotional Intelligence * Cultural Intelligence | + | Darvishmotevali et al. (2018)**.** |
|  | Hedonic Well-Being * Direct Achieving Style | + | Zhang & Zhao (2021). |
|  | Hedonic Well-Being * Instrumental Achieving Style | + | Zhang & Zhao (2021). |
|  | Negative Affect – OCB-Is – CP | + | Chuang et al. (2019). |
|  | Negative Affect * Creative Self-Efficacy | + | Thundiyil et al. (2016)**.** |
|  | Positive Affect * Negative Affect | + | Gong & Zhang (2017). |
|  | Positive Affect Change * Negative Affect Change | - | Gong & Zhang (2017). |
|  | Positive Emotion * Perceived Organizational Support | + | Qian & Jiang (2023). |
|  | Positive Affect * Creative Self-Efficacy | - | Thundiyil et al. (2016)**.** |
|  | Positive Affect * Negative Affect * Creative Self-Efficacy | - | Thundiyil et al. (2016)**.** |
| Cognitive Ability | Cultural Intelligence * Task Conflict | - | Hu et al. (2019)**.** |
|  | Cultural Intelligence * Relationship Conflict | + | Hu et al. (2019)**.** |
|  | Innovative Cognitive Style - Feedback Inquiry – CP | + | De Stobbeleir et al. (2011). |
| Others | Diversity Ideology * Design for Autonomy | + | Cheung & Zhang (2021). |
|  | Diversity Ideology * Interactive Office Design | + | Cheung & Zhang (2021). |
|  | Perceived Organizational Support – Feedback Inquiry – CP | + | De Stobbeleir et al. (2011). |
|  | Prohibitive Voice Behavior – Psychosocial Prosperity – CP | + | Prince & Rao (2022). |
|  | Prohibitive Voice Behavior – Psychosocial Prosperity * Work Alienation – CP | - | Prince & Rao (2022). |
|  | Promotive Voice Behavior – Psychosocial Prosperity – CP | + | Prince & Rao (2022). |
|  | Promotive Voice Behavior – Psychosocial Prosperity * Work Alienation – CP | - | Prince & Rao (2022). |
|  | Psychological Capital – Trust in Organization – CP | + | Ozturk & Karatepe (2019). |
|  | Voice Behavior * Challenge Stressors | - | Song et al. (2017)**.** |
|  | Voice Behavior * Hindrance Stressors | + | Song et al. (2017)**.** |

*Note: Relationship (R): positive (+); or negative. CP: Creative Performance.*
